# Risk of New-Onset Postoperative Depression After Holmium Laser Enucleation Versus Transurethral Resection of the Prostate for Benign Prostatic Hyperplasia: A Nationwide Retrospective Cohort Study

**DOI:** 10.3390/jcm15155992

**Published:** 2026-08-01

**Authors:** Won Jae Kim, So Hyeon Gwon, Dongu Lee, Eun Lee, Nak-Hoon Son, Woo Jung Kim

**Affiliations:** 1 Institute of Behavioral Science in Medicine, Yonsei University College of Medicine, Seoul 03722, Republic of Korea; kwj224@yuhs.ac (W.J.K.); leeeun@yuhs.ac (E.L.); 2 Department of Psychiatry, Severance Hospital, Yonsei University College of Medicine, Seoul 03722, Republic of Korea; 3 Department of Statistics, Keimyung University, Daegu 42601, Republic of Korea; gwonsohyeon@kmu.kr; 4 Department of Urology, Hana General Hospital, Cheongju 28378, Republic of Korea; hana11822@cjhana.com; 5 Yonsei Institute for Digital Health, Yonsei University, Seoul 03722, Republic of Korea; 6 Department of Psychiatry, Yongin Severance Hospital, Yonsei University College of Medicine, Yongin 16995, Republic of Korea; 7 Yonsei Graduate Program in Cognitive Science, Yonsei University, Seoul 03722, Republic of Korea

**Keywords:** prostatic hyperplasia, postoperative complications, depression, cohort studies, risk factors

## Abstract

**Background/Objectives:** Depression is a frequently overlooked but clinically important complication following surgical treatment of benign prostatic hyperplasia (BPH). This study aimed to compare the risk of new-onset postoperative depression following transurethral resection of the prostate (TURP) and holmium laser enucleation of the prostate (HoLEP) in patients with BPH. **Methods:** We conducted a retrospective cohort study using the Korean Health Insurance Review and Assessment database. Patients diagnosed with BPH between 2013 and 2022 who underwent TURP or HoLEP and had no prior diagnosis of a psychiatric disorder were included (*n* = 30,878 and *n* = 21,521, respectively). The primary outcome was a diagnosis of depression within one year after surgery. Multivariable logistic regression and propensity score matching were used to adjust for covariates, including age, comorbidities, medication use, geographic region, and hospital level. Sensitivity analyses were conducted using follow-up periods of one, three, six, and nine months. **Results:** In the unadjusted model, HoLEP was associated with higher odds of postoperative depression compared with TURP (odds ratio [OR] = 1.338, 95% confidence interval [CI]: 1.245–1.438; *p* < 0.0001). This association remained significant after multivariable adjustment (OR = 1.350, 95% CI: 1.255–1.451; *p* < 0.0001) and propensity score matching (OR = 1.364, 95% CI: 1.259–1.476; *p* < 0.0001). The association was observed across alternative follow-up windows in the sensitivity analyses. **Conclusions:** HoLEP is associated with higher odds of new-onset postoperative depression compared with TURP. These findings may inform perioperative mental health assessment for patients undergoing surgical treatment for BPH, and warrant further evaluation to establish causality or clarify the underlying mechanism.

## 1. Introduction

Benign prostatic hyperplasia (BPH) is a growing urologic problem among men. A recent report showed 1125.02 prevalent cases per 100,000 individuals in 2021—more than double the prevalence in 1990 [1]—and current projections expect that this number will continue to rise [2]. BPH is known to affect patient quality of life through the discomfort of lower urinary tract symptoms, such as urinary frequency and nocturia [3]. Moreover, the indirect burden of BPH on patient families and society is expected to increase, especially because of the rapidly aging population [3,4]. In addition to symptoms that are directly caused by enlarged prostate, the condition can also lead to other complications, such as recurrent urinary tract infections, erectile dysfunction, or even renal dysfunction [3,5]. Hence, an optimal treatment strategy that effectively addresses both the symptoms and complications of BPH is becoming increasingly important.

Depression is an often overlooked complication of BPH and its surgical treatment. Previous studies have shown that depressive symptoms are present in over 20% of BPH patients [6], and that depression is significantly more prevalent in this population compared with people without BPH or lower urinary tract symptoms [7]. A 2021 cohort study revealed that depression remains a serious issue even after surgical treatment, as 24.9% of patients reportedly suffered from mild to severe depression after transurethral resection of prostate (TURP) [8]. This postoperative depression was related not only to patient preoperative status, such as the duration of BPH, but also to their postoperative symptoms, including lower urinary tract symptoms and incontinence [8]. Considering that postoperative depression after major surgeries is reportedly associated with various morbidities and mortalities [9], it is important to take this complication more seriously in the treatment of BPH.

Holmium laser enucleation of the prostate (HoLEP), in which the prostate is enucleated with a laser and then pushed into the bladder to be morcellated [10], has recently garnered attention for its greater efficacy compared with TURP, which has long been considered the gold standard [11]. Several benefits of using HoLEP instead of TURP have been consistently reported, including better recovery of urinary function, shorter catheterization and bladder irrigation time, shorter hospital stays, and a less frequent need for blood transfusion [12,13]. HoLEP is also reported to lead to fewer surgery-related complications, such as hyponatremia or urethral stricture [12]. Given these merits, surgeons are increasingly using HoLEP as surgical treatment of BPH [14].

However, postoperative depression following HoLEP has not been properly studied or compared with TURP. Most preexisting studies that compare HoLEP with TURP have largely focused on postoperative urinary function, surgical complications and the length of hospital stay [12,13], whereas nationwide database-based comparisons of postoperative mental health outcomes remain limited. This gap in the literature is notable because psychological outcomes of surgery may not necessarily improve in parallel with conventional surgical outcomes, but may also be affected by other factors, including residual postoperative symptoms and unmet patient expectations [8,15]. Specifically, the differences between TURP and HoLEP in postoperative urinary incontinence, irritative urinary symptoms, surgical complexity and opportunities for diagnosis may contribute to a differential risk of postoperative depression between the two surgeries. Further exploration of the incidence of postoperative depression after HoLEP and its differential risk compared with TURP could improve the physical and psychological recovery of BPH patients after surgical treatment. In this study, we aimed to analyze and compare the incidence of new-onset postoperative depression following TURP and HoLEP for the treatment of BPH.

## 2. Materials and Methods

### 2.1. Data Source and Study Design

This retrospective observational study used regression analysis to compare the odds of being diagnosed with postoperative depression after surgical treatment of BPH. We used the Korean Health Insurance Review and Assessment (HIRA) database for this study (Project number: M20230810001), which contains information for every patient covered by Korea’s national health insurance, which is approximately 98% of the total population [16]. The HIRA database collects diagnostic and treatment information, as well as socioeconomic details such as age and sex [16].

### 2.2. Participant Selection

**Figure 1:**
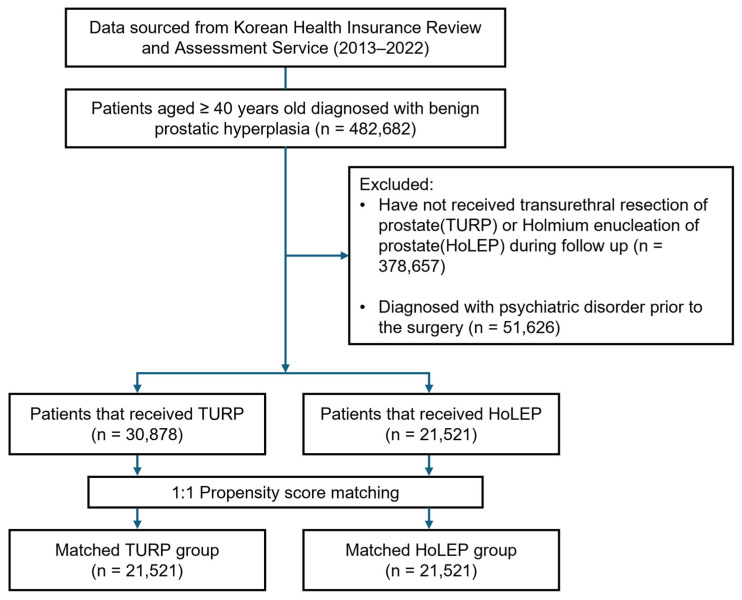
Flow chart of study participants. HoLEP = holmium laser enucleation of the prostate; TURP = transurethral resection of the prostate.

We included patients who had a diagnosis of BPH from 2013 to 2022, designated by an International Classification of Diseases 10th Revision (ICD-10) code of N40, N40.1, N40.2, N40.3, or N40.8. From those patients, we excluded those who did not undergo TURP or HoLEP (surgery codes R3975 and R3977, respectively) and those with a diagnosed psychiatric disorder (any ICD-10 F code), before the time of surgery. After applying all the inclusion and exclusion criteria, 30,878 participants were included in the TURP group, and 21,521 participants were included in the HoLEP group. A schematic of the participant selection is shown in Figure 1.

### 2.3. Variables and Outcome

Information on the participants was collected at baseline, defined as the time of surgery. Patient age was included as a continuous variable, whereas patient sex and medical history conditions were included as dichotomous variables. We specifically considered conditions that could affect BPH and depression from patient medical history, namely hypertension (ICD-10 codes: I10–I13, I15), diabetes (E10–E14), dyslipidemia (E780–E785), heart failure (I50), arrhythmia (I47–I49), cerebrovascular disease (I60–I69), thyroid disease (E00–E07), and chronic obstructive pulmonary disease (J43.1, J43.2, J43.8, J43.9, J44). Androgenetic alopecia (L64) was also included, as this condition is androgen-dependent, like BPH, and reportedly associated with prostate size [17,18].

We considered patient history of taking drugs that could affect the outcome of the analysis, specifically tricyclic antidepressants (TCAs), benzodiazepines, and zolpidem, which can be prescribed to urology patients even without a psychiatric diagnosis. We also included the use of 5-alpha reductase inhibitors (5-ARIs) or alpha blockers, both pharmacological treatment options for BPH, because they can affect hormone levels and prostate size and may indirectly represent the severity of BPH symptoms or prostate size before surgery [19,20].

Using the gathered medical history data, we calculated a Charlson comorbidity index (CCI) for each patient to quantify the degree of comorbidity [21]. The variables used for the CCI calculation are shown in Table S1. The CCI was analyzed as a continuous variable for baseline comparisons, but was categorized into three groups (0–2, 3–4, and ≥5) for regression analyses.

To account for potential biases related to geographic location and hospital capacity, we included both region and hospital level as independent variables in our analysis. Hospitals located in metropolitan cities were classified as urban, whereas all others were categorized as rural. Hospitals designated as secondary and tertiary care institutions were classified together as higher-level hospitals, reflecting their greater complexity and capacity for specialized care relative to primary hospitals; this variable was analyzed dichotomously in the regression models.

The primary outcome was a diagnosis of depression (F32 or F33) within 1 year after surgery. All participants were followed until 1 year after surgery, diagnosis of depression, or the end of data availability (31 December 2022), whichever occurred first.

### 2.4. Statistical Analysis

Continuous variables were presented as mean and standard deviation, and comparisons between the two surgical groups were performed using an independent two-sample *t*-test. Categorical variables were presented as frequencies and percentages, and differences between the two groups were analyzed using the chi-square test.

To determine whether the occurrence of depression depended on the surgical method, we performed binary logistic regression analysis. Because underlying diseases or medications may also influence the occurrence of depression, we performed a multivariate logistic regression analysis, including statistically significant variables at a significance level of 0.05 as adjusted factors. The analysis results were presented as odds ratios (ORs) and 95% confidence intervals (CIs).

In retrospective studies, imbalances in baseline characteristics can cause bias in the results, so propensity score matching (PSM) was performed to reduce this bias [22]. The propensity score was calculated using logistic regression analysis based on variables that were statistically significant at the 0.05 level among the observed covariates at baseline. Subsequently, 1:1 matching was performed between subjects with the same scores to ensure an equal number of subjects in each group and to maintain balance among covariates, thereby minimizing the influence of confounding variables. Balance was assessed based on the standardized mean difference (SMD), with values below 0.1 considered statistically balanced [23].

A sensitivity analysis was performed across multiple follow-up windows to examine whether the timing of depressive symptom manifestation or diagnosis influenced the observed association. Specifically, diagnoses of depression that occurred within 1, 3, 6, or 9 months after surgery were defined as separate dependent variables to examine the consistency and reliability of depressive symptoms after surgery. All analyses were performed using SAS software version 9.4 (SAS Institute Inc., Cary, NC, USA).

## 3. Results

### 3.1. Baseline Characteristics

The baseline characteristics of the study participants are shown in Table 1. Patients in the TURP group were significantly older than those in the HoLEP group. There were significant differences between groups in the prevalence of every condition in the participants’ medical history, except arrhythmia and androgenetic alopecia. When the CCI was calculated and compared between groups, that of the TURP group was significantly higher than that of the HoLEP group. The TURP group had a significantly higher usage of TCAs, and the HoLEP group was significantly more likely to be taking alpha blockers or 5-ARIs. Lastly, patients who underwent HoLEP were significantly more likely to have had the surgery in higher-level hospitals and to live in an urban area. After performing PSM, the SMDs for all covariates were found to be less than 0.1 (Figure S1), indicating that the baseline characteristics between the two groups were well balanced after matching.

### 3.2. Difference in Postoperative Depression Following TURP and HoLEP

In the unmatched crude analysis, new-onset depression within 12 months after surgery occurred in 1651 of 30,878 patients (5.35%) in the TURP group and 1512 of 21,521 patients (7.03%) in the HoLEP group, corresponding to a risk difference of 1.68 percentage points (95% CI, 1.26–2.10). After propensity score matching, 1130 of 21,521 patients (5.25%) in the TURP group and 1512 of 21,521 patients (7.03%) in the HoLEP group developed depression within 12 months, yielding a risk difference of 1.78 percentage points (95% CI, 1.32–2.23).

**Figure 2:**
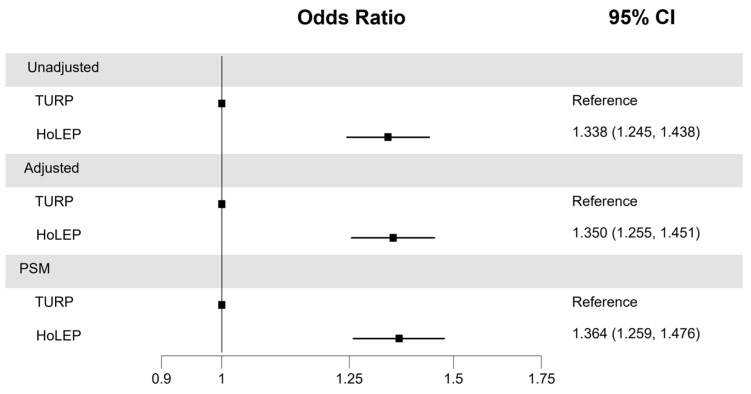
Forest plot of multivariate logistic regression for onset of depression at 1 year after surgery.

The results of the logistic regression analyses are shown in Table 2 and Figure 2. In the unadjusted model, participants in the HoLEP group had significantly higher odds of being diagnosed with postoperative depression (OR = 1.338, *p* < 0.0001). The independent variables that showed significant associations in the unadjusted model (i.e., all the variables except androgenetic alopecia and the region of hospital) were adjusted in the multivariate model. In this model, the significant association between the surgical method and odds of postoperative depression diagnosis was maintained (OR = 1.350, *p* < 0.0001). This was also true when the analysis was carried out using the PSM dataset (OR = 1.364, *p* < 0.0001).

### 3.3. Sensitivity Analysis

Odds ratios for postoperative depression diagnosis were calculated at different timepoints for sensitivity analysis, and the results are shown in Table 3. In addition to our main findings from 1 year after surgery, HoLEP patients had consistently higher odds of being diagnosed with postoperative depression at 1, 3, 6, and 9 months. These results were consistent for the multivariate model and for participants after PSM.

## 4. Discussion

In this study, we investigated and compared the risk of new-onset postoperative depression among patients undergoing TURP and HoLEP for BPH. Our results show that HoLEP patients had higher odds of being diagnosed with depression after surgery compared with TURP patients, and this difference persisted after model adjustment and PSM, including comorbidities and hospital level as variables. To our knowledge, this is the first study to analyze the risk of postoperative depression following HoLEP and compare it with the risk following TURP.

Depression is an important consideration in postoperative care due to its relationship with postoperative pain, health-related quality of life, and mortality [9]. Patients who undergo TURP have been reported to be vulnerable to depression, with one study showing that a quarter of patients experienced postoperative depression after TURP [8]. Although HoLEP is a promising alternative to TURP in terms of surgical outcomes and complications, our results show even higher odds of postoperative depression following HoLEP compared with TURP. Clinicians could benefit from considering this psychiatric complication in BPH patient postoperative care.

Several candidate mechanisms, which could not be directly assessed using claims data, may help explain the increased risk of depression following HoLEP compared with TURP. The most clinically apparent and observable explanation involves postoperative urinary symptoms. Although HoLEP is reportedly superior in many ways, studies have shown that it can lead to higher prevalence of some symptoms, such as postoperative dysuria and incontinence [12,24,25]. Considering that the severity of lower urinary tract symptoms and urinary incontinence were reported to be associated with depression after surgery for BPH [8], their contribution to psychological distress and reduced quality of life may attenuate the benefits of HoLEP in terms of vulnerability to postoperative depression.

Second, the operative burden of HoLEP could also be a contributing factor. HoLEP is generally associated with greater surgical complexity and longer operative times compared with TURP [24,25]. A recent population-scale retrospective cohort analysis found that a longer operative duration was significantly associated with greater incidence of postoperative depression and anxiety [26], and similar findings were observed even when the type of surgery was limited to urology [27]. Although clinical trials showed favorable outcomes for HoLEP, its complexity and steep learning curve can lead to perioperative and long-term complications in real-world settings that could affect patient psychological wellbeing [28].

Though this last potential cause is speculative due to limited preexisting evidence, psychological factors such as preoperative anxiety and patient expectations could be relevant to the risk of postoperative depression. HoLEP is a newer surgical method that involves more extensive removal of the prostate, which could lead patients to have higher expectations of symptom resolution following HoLEP compared with TURP. As patients may still experience residual BPH symptoms and bladder dysfunction following HoLEP [25,29], these symptoms may cause depression due to unmet expectations [30]. Previous studies have shown that providers do not consistently give HoLEP patients the proper information about possible postoperative symptoms [31], and that dissatisfaction after BPH surgery may lead to surgery decision regrets [15]. Furthermore, the fact that HoLEP is more extensive by nature and involves a longer operation time may cause a higher level of preoperative psychological disturbance before surgery, which has been reported as a predictor of postoperative worsening of psychiatric symptoms [32]. Although direct comparative data on preoperative anxiety and expectations between HoLEP and TURP are limited, these psychosocial factors may differentially affect patients undergoing HoLEP and could partly contribute to the higher odds of postoperative depression observed in this group. Further studies incorporating these psychological factors are warranted.

We implemented various follow-up windows as a sensitivity analysis to account for possible delays in the manifestation of depressive symptoms or diagnosis of postoperative depression. Patients who underwent HoLEP were at significantly higher risk of postoperative depression at each follow-up in both the adjusted model and the PSM model, but the OR steadily decreased with longer follow-up periods. This may suggest that differences in postoperative symptoms or unmet patient expectations are experienced early in the postoperative phase, since studies have shown that residual or even worsening lower urinary tract symptoms can be identified as early as one month after surgery [33]. However, similar results could also arise from other factors, such as perioperative coding, recording of previously undiagnosed depression, and more frequent early postoperative visits and monitoring. Future studies that utilize the number of postoperative visits as a variable or include a landmark analysis would be beneficial in clarifying whether the observed association in our results reflects a genuine psychiatric consequence of surgery.

The results of our analysis of baseline characteristics show significant differences in patient age and preexisting comorbidities. TURP patients were significantly older and had a higher prevalence of most comorbidities, except for arrhythmia and androgenetic alopecia. This finding aligns with a previous study that compared TURP and laser prostatectomy in the United States, which showed that TURP was more common than laser prostatectomy in older patients and those with more comorbidities [34]. Moreover, in our study, HoLEP patients were more likely to be treated in higher-level or urban hospitals. According to an analysis of BPH surgeries in Korea in 2019, approximately 80% of TURP and HoLEP procedures were performed in superior general hospitals or general hospitals, and a majority were performed in Seoul, the largest city in Korea [35]. Our results show that HoLEP was more strongly aligned with this trend. To account for these differences in baseline characteristics, we implemented PSM to eliminate any potential bias. After matching, the SMDs for all independent variables were within ±0.1, indicating no imbalances between the groups [23]. The significant difference in the onset of postoperative depression between the groups was maintained after PSM, which further supports the robustness of our results.

Our regression analysis results highlight several other important risk factors for postoperative depression. We found that older age, cardiometabolic and chronic systemic comorbidities, and higher CCI scores were associated with elevated risk, consistent with previous studies [36,37]. Usage of alpha blockers or 5-ARIs at the time of surgery was also associated with increased risk of postoperative depression. Use of these medications may indirectly imply more severe BPH symptoms or a larger preoperative prostate volume [19,20]. Notably, the results of a previous study demonstrated an association between use of alpha blockers or 5-ARIs and a higher risk of depression [38]. In our study, patients who were taking TCAs, benzodiazepines, or zolpidem were also at higher risk of developing postoperative depression. Although benzodiazepines and zolpidem may themselves increase the risk of depression [39,40], our finding may also be explained by undiagnosed psychiatric problems of the medication users. Lastly, having surgery in a higher-level hospital was associated with a higher prevalence of postoperative depression. This result could be attributed to referral bias or detection bias, because secondary and tertiary hospitals handle cases with more complex or severe presentations and have a more robust screening process for comorbidities.

One strength of our study is our use of the HIRA database, which enabled us to analyze data from over 52,000 patients, including information on various confounders. Furthermore, because this nationwide health insurance database covers most of the Korean population, the potential bias due to selective recruitment was minimal. Second, we used model adjustment, PSM, and a sensitivity analysis to further explore our results and support their robustness. Finally, by considering the hospital level and region, we accounted for socioeconomic confounding and potential detection bias.

The most important limitation of our study is that variables that may have been beneficial for our analysis were unavailable, due to the inherent limitations of health insurance claims data. First, preoperative status, including prostate size and urological symptoms, was not used in our analysis. Although we used variables such as the intake of BPH-related medication, hospital level and region to reduce selection bias as much as possible, prostate size and symptom severity could be an important unmeasured confounder, since HoLEP is known to be more preferred in larger and more symptomatic glands [41]. Second, factors regarding the surgery itself, such as the length of operation or the mode of anesthesia or sedatives used, could not be included. Third, while perioperative urological symptoms collected using tests and self-reported questionnaires have been reported to be useful in postoperative quality-of-outcome assessment [42], these were also unavailable in our data. There were also additional analyses that could be helpful in the interpretation of our results that we could not perform due to the limitations of claims data. While the data had information on the hospital level and region that we used in our analysis, it did not provide any hospital identifiers, which prohibited us from matching patients from the same hospital or limiting the dataset to only include hospitals that perform both surgeries. Also, since information on patient death was limited to in-hospital deaths, we were unable to perform a time-to-event analysis using death as a competing risk. Further studies that incorporate these variables to yield data-supported results would be beneficial in discovering the mechanism behind our results.

In addition to these data-related constraints, several methodological limitations should also be noted. As this was a retrospective observational study, we were unable to identify the causality or the mechanism of the association between postoperative depression and the surgical methods. Also, although the HIRA database is useful in its coverage and the volume of data, we could only use ICD-10 codes to determine our variables and outcomes, which might have caused misclassification or underdiagnosis. Requiring repeat visits or prescription of antidepressants as part of the outcome definition would have increased the specificity of our results. Finally, we did not include the calendar year of surgery as a covariate, even though temporal changes over the study period may have influenced the choice of surgery or the rate of diagnosis of postoperative depression. Further studies that improve on these limitations are warranted.

In conclusion, our results showed that HoLEP was associated with a higher rate of recorded diagnoses of postoperative depression in claims data, even after accounting for various biases or confounders. Further studies are needed to determine whether this association reflects the surgical procedure itself, postoperative symptom burden, detection bias, or unmeasured confounding.

## Figures and Tables

**Table 1:** Characteristics of participants at the time of surgery.

Characteristics	Full Data (Unadjusted) (*n* = 52,399)				PSM 1:1 (*n* = 43,042)			
	TURP (*n* = 30,878)	HoLEP (*n* = 21,521)	*p*-Value	SMD	TURP (*n* = 21,521)	HoLEP (*n* = 21,521)	*p*-Value	SMD
Age, mean (SD)	70.04 (8.70)	69.45 (7.84)	<0.0001	0.072	69.51 (8.43)	69.45 (7.84)	0.3965	0.008
Hypertension, No. (%)			0.0436	0.018			0.4289	0.008
No	12,745 (41.28)	9073 (42.16)			8992 (41.78)	9073 (42.16)		
Yes	18,133 (58.72)	12,448 (57.84)			12,529 (58.22)	12,448 (57.84)		
Diabetes, No. (%)			0.0239	0.020			0.6664	−0.004
No	17,846 (57.80)	12,651 (58.78)			12,695 (58.99)	12,651 (58.78)		
Yes	13,032 (42.20)	8870 (41.22)			8826 (41.01)	8870 (41.22)		
Dyslipidemia, No. (%)			<0.0001	−0.040			0.3831	−0.008
No	13,193 (42.73)	8776 (40.78)			8865 (41.19)	8776 (40.78)		
Yes	17,685 (57.27)	12,745 (59.22)			12,656 (58.81)	12,745 (59.22)		
Heart failure, No. (%)			0.0133	0.022			0.0030	−0.028
No	28,695 (92.93)	20,119 (93.49)			20,267 (94.17)	20,119 (93.49)		
Yes	2183 (7.07)	1402 (6.51)			1254 (5.83)	1402 (6.51)		
Arrhythmia, No. (%)			0.1305	0.013			0.5912	−0.005
No	28,128 (91.09)	19,686 (91.47)			19,717 (91.62)	19,686 (91.47)		
Yes	2750 (8.91)	1835 (8.53)			1804 (8.38)	1835 (8.53)		
Cerebrovascular disease, No. (%)			0.0349	0.019			0.4618	−0.007
No	26,105 (84.54)	18,339 (85.21)			18,393 (85.47)	18,339 (85.21)		
Yes	4773 (15.46)	3182 (14.79)			3128 (14.53)	3182 (14.79)		
Thyroid disease, No. (%)			<0.0001	−0.041			0.0467	−0.019
No	24,990 (80.93)	17,067 (79.30)			17,233 (80.08)	17,067 (79.30)		
Yes	5888 (19.07)	4454 (20.70)			4288 (19.92)	4454 (20.70)		
Androgenetic alopecia, No. (%)			0.2414	−0.012			0.3360	−0.008
No	30,846 (99.90)	21,491 (99.86)			21,498 (99.89)	21,491 (99.86)		
Yes	32 (0.10)	30 (0.14)			23 (0.11)	30 (0.14)		
COPD, No. (%)			<0.0001	0.044			0.0934	−0.016
No	26,404 (85.51)	18,725 (87.01)			18,841 (87.55)	18,725 (87.01)		
Yes	4474 (14.49)	2796 (12.99)			2680 (12.45)	2796 (12.99)		
Alpha blocker or 5-ARI, No. (%)			0.0077	−0.024			0.0208	−0.022
No	23,171 (75.04)	15,928 (74.01)			16,137 (74.98)	15,928 (74.01)		
Yes	7707 (24.96)	5593 (25.99)			5384 (25.02)	5593 (25.99)		
Tricyclic antidepressants, No. (%)			0.0409	0.018			0.5388	−0.006
No	29,993 (97.13)	20,968 (97.43)			20,988 (97.52)	20,968 (97.43)		
Yes	885 (2.87)	553 (2.57)			533 (2.48)	553 (2.57)		
Benzodiazepine, No. (%)			0.3640	0.008			0.8495	0.002
No	24,343 (78.84)	17,037 (79.16)			17,021 (79.09)	17,037 (79.16)		
Yes	6535 (21.16)	4484 (20.84)			4500 (20.91)	4484 (20.84)		
Zolpidem, No. (%)			0.0988	0.014			0.7371	0.003
No	30,300 (98.13)	21,160 (98.32)			21,151 (98.28)	21,160 (98.32)		
Yes	578 (1.87)	361 (1.68)			370 (1.72)	361 (1.68)		
CCI, mean (SD)	2.80 (2.41)	2.69 (2.19)	<0.0001	0.049	2.65 (2.26)	2.69 (2.19)	0.1148	−0.015
Region, No. (%)			<0.0001	−0.269			0.8140	−0.002
Rural	12,661 (41.00)	6093 (28.31)			6115 (28.41)	6093 (28.31)		
Urban	18,217 (59.00)	15,428 (71.69)			15,406 (71.59)	15,428 (71.69)		
Hospital level, No. (%)			<0.0001	−0.072			0.2502	0.011
Primary	7086 (22.95)	4301 (19.99)			4206 (19.54)	4301 (19.99)		
Higher-level	23,792 (77.05)	17,220 (80.01)			17,315 (80.46)	17,220 (80.01)		

*p*-Value was calculated using chi-square tests for categorical variables and independent t-test for age and Charlson Comorbidity Index. 5-ARI = 5α-reductase inhibitor; CCI = Charlson comorbidity index; COPD = chronic obstructive pulmonary disease; PSM = propensity score matching; SD = standard deviation; SMD = standardized mean difference.

**Table 2:** Logistic regression for onset of depression at 1 year after surgery.

Variables	Unadjusted		Adjusted		After PSM	
	OR (95% CI)	*p*-Value	OR (95% CI)	*p*-Value	OR (95% CI)	*p*-Value
Surgery						
TURP	Reference		Reference		Reference	
HoLEP	1.338 (1.245–1.438)	<0.0001	1.350 (1.255–1.451)	<0.0001	1.364 (1.259–1.476)	<0.0001
Age						
<65 years	Reference		Reference			
≥65 years	1.544 (1.411–1.690)	<0.0001	1.410 (1.284–1.547)	<0.0001		
Hypertension						
No	Reference		Reference			
Yes	1.120 (1.040–1.205)	0.0026	0.960 (0.884–1.042)	0.3271		
Diabetes						
No	Reference		Reference			
Yes	1.203 (1.119–1.293)	<0.0001	1.121 (1.031–1.219)	0.0074		
Dyslipidemia						
No	Reference		Reference			
Yes	1.087 (1.010–1.170)	0.0254	0.952 (0.873–1.037)	0.2590		
Heart failure						
No	Reference		Reference			
Yes	1.276 (1.120–1.454)	0.0002	1.077 (0.931–1.246)	0.3191		
Arrhythmia						
No	Reference		Reference			
Yes	1.196 (1.061–1.347)	0.0033	1.048 (0.923–1.189)	0.4703		
Cerebrovascular disease						
No	Reference		Reference			
Yes	1.435 (1.311–1.571)	<0.0001	1.281 (1.164–1.409)	<0.0001		
Thyroid disease						
No	Reference		Reference			
Yes	1.104 (1.011–1.205)	0.0279	0.994 (0.904–1.093)	0.9005		
Androgenetic alopecia						
No	Reference					
Yes	1.076 (0.391–2.963)	0.8872				
COPD						
No	Reference		Reference			
Yes	1.245 (1.129–1.372)	<0.0001	1.088 (0.984–1.203)	0.1005		
Alpha blocker or 5-ARI						
No	Reference		Reference			
Yes	1.154 (1.065–1.251)	0.0005	0.981 (0.901–1.067)	0.6557		
Tricyclic antidepressants						
No	Reference		Reference			
Yes	1.640 (1.369–1.965)	<0.0001	1.476 (1.229–1.773)	<0.0001		
Benzodiazepine						
No	Reference		Reference			
Yes	1.319 (1.214–1.432)	<0.0001	1.245 (1.144–1.354)	<0.0001		
Zolpidem						
No	Reference		Reference			
Yes	2.149 (1.759–2.627)	<0.0001	1.873 (1.524–2.301)	<0.0001		
CCI						
CCI = 0~2	Reference		Reference			
CCI = 3~4	1.100 (1.013–1.196)	0.6847	0.997 (0.909–1.092)	0.8612		
CCI = 5 or higher	1.251 (1.140–1.373)	<0.0001	1.008 (0.897–1.133)	0.8536		
Region						
Rural	Reference					
Urban	1.022 (0.948–1.102)	0.5655				
Hospital level						
Primary	Reference		Reference			
Higher-level	1.558 (1.412–1.720)	<0.0001	1.430 (1.292–1.581)	<0.0001		

CI = confidence interval; OR = odds ratio.

**Table 3:** Sensitivity analysis of postoperative depression across different follow-up periods.

Follow-Up Period	Surgery	Unadjusted		Adjusted		After PSM	
		OR (95% CI)	*p*-Value	OR (95% CI)	*p*-Value	OR (95% CI)	*p*-Value
1 month	TURP	Reference		Reference		Reference	
	HoLEP	2.794 (2.363–3.304)	<0.0001	2.784 (2.354–3.294)	<0.0001	2.880 (2.377–3.490)	<0.0001
3 months	TURP	Reference		Reference		Reference	
	HoLEP	2.576 (2.277–2.913)	<0.0001	2.523 (2.228–2.857)	<0.0001	2.613 (2.272–3.004)	<0.0001
6 months	TURP	Reference		Reference		Reference	
	HoLEP	1.697 (1.554–1.853)	<0.0001	1.725 (1.578–1.885)	<0.0001	1.782 (1.613–1.967)	<0.0001
9 months	TURP	Reference		Reference		Reference	
	HoLEP	1.468 (1.357–1.588)	<0.0001	1.479 (1.367–1.601)	<0.0001	1.517 (1.390–1.656)	<0.0001

## Data Availability

Restrictions apply to the availability of these data. Data were obtained from Korean Health Insurance Review and Assessment Service and are available from the authors with the permission of Korean Health Insurance Review and Assessment Service.

## Supplementary Materials

The following supporting information can be downloaded at: https://www.mdpi.com/article/10.3390/jcm15155992/s1, Table S1: List of variables used for the calculation of Charlson comorbidity index; Figure S1: Standardized mean differences of all variables before and after matching.

## Abbreviations

The following abbreviations are used in this manuscript:

BPH	Benign prostatic hyperplasia
TURP	Transurethral resection of the prostate
HoLEP	Holmium laser enucleation of the prostate
OR	Odds ratio
CI	Confidence interval
HIRA	Health Insurance Review and Assessment
ICD-10	International Classification of Diseases 10th Revision
TCAs	Tricyclic antidepressants
5-ARI	5-alpha reductase inhibitors
CCI	Charlson comorbidity index
PSM	Propensity score matching
SMD	Standardized mean difference
