# Supplementary material for: Risk of New-Onset Postoperative Depression After Holmium Laser Enucleation Versus Transurethral Resection of the Prostate for Benign Prostatic Hyperplasia: A Nationwide Retrospective Cohort Study
Source: J Clin Med. 2026 Aug 1;15(15):5992. doi: 10.3390/jcm15155992 (PMC13467408; doi:10.3390/jcm15155992)
Supplement: Supplementary file 1 [file jcm-15-05992-s001.zip › jcm-4443441-supplementary.pdf]

Table S1. List of variables used for the calculation of Charlson comorbidity index

Figure S1. Standardized mean differences of all variables before and after matching

Table S1. List of variables used for the calculation of Charlson comorbidity index

Type of disease	Score	International Classification of Diseases 10th Revision
Chronic pulmonary disease	1	I278, I279, J4, J60, J61, J62, J63, J64, J65, J66, J67, J684, J701, J703
Rheumatic disease	1	M05, M06, M315, M32, M33, M34, M351, M353, M360
Diabetes with chronic complication	1	E102, E103, E104, E105, E107, E112, E113, E114, E115, E117, E122, E123, E124, E125, E127, E132, E133, E134, E135, E137, E142, E143, E144, E145, E147
Renal disease	1	I120, I131, N032, N033, N034, N035, N036, N037, N052, N053, N054, N055, N056, N057, N18, N19, N250, Z49, Z940, Z992
Congestive heart failure	2	I099, I110, I130, I132, I255, I420, I425, I426, I427, I428, I429, I43, I50, P290
Dementia	2	F00, F01, F02, F03, F051, G30, G311
Mild liver disease	2	B18, K700, K701, K702, K703, K709, K713, K714, K715, K717, K73, K74, K760, K762, K763, K764, K768, K769, Z944
Hemiplegia or paraplegia	2	G041, G114, G801, G802, G81, G82, G830, G831, G832, G833, G834, G839
Any malignancy, including lymphoma and leukemia, except malignant neoplasm of skin	2	C0, C1, C2, C3, C40, C41, C43, C45, C46, C47, C48, C49, C5, C6, C70, C71, C72, C73, C74, C75, C76, C81, C82, C83, C84, C85, C88, C9
Moderate or severe liver disease	4	I85, I864, I982, K704, K711, K721, K729, K765, K766, K767
Acquired immunodeficiency syndrome / human immunodeficiency virus	4	B20, B21, B22, B24
Metastatic solid tumor	6	C77, C78, C79, C80

Figure S1. Standardized mean differences of all variables before and after matching

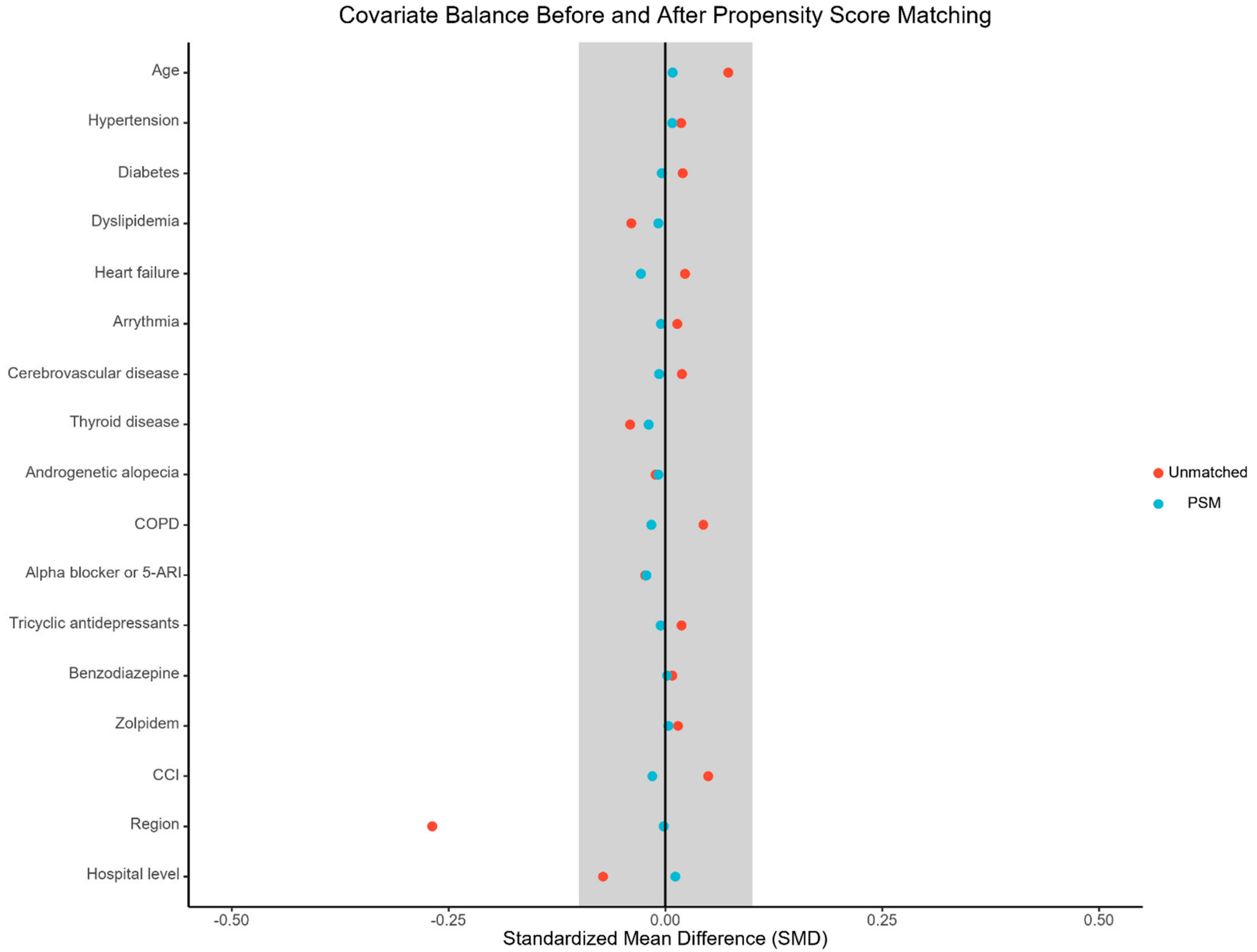

5-ARI = 5-alpha reductase inhibitor; CCI = Charlson comorbidity index; COPD = chronic obstructive pulmonary disease; PSM = propensity score matching.
